# Dr. George Gregory's Elements of the Theory and Practice of Physic

**Published:** 1820-12-01

**Authors:** 


*Elements of the Theory and Practice of Physic, designed for the Use of Students. Part 1, including the Symptoms, Pathology, and Treatment of Diseases.* By George Gregory, M. D. Licentiate of the Royal College of Physicians, and Senior Physician to the Saint George's and Saint James's Infirmary. Octavo, pp. 402, Burgess & Co. London, 1820.

It requires some genius to compose—but it requires much judgment to compile. Systems of physic may be either original, instance Cullen, Darwin, Brown—or compiled, instance that Koran of medical faith, "Thomas's Practice." In our compilations we have generally a reference to, or quotations from, a host of authors, bearing the most contradictory testimony on every subject; one disadvantage attendant on which is, that the inexperienced is much puzzled which theory to believe, or which practice to pursue. There is yet another course, namely, an *eclectic* one, in which the writer adopts what he thinks best from others, or from his own observations, and thus lays down, as it were, but one system of pathology, and one line of practice. The author of a book of this kind, voluntarily incurs a most serious responsibility, inasmuch as the student or practitioner, placing faith in his preceptor's judgment, adopts implicitly his code of instructions. If a work of this kind be ably executed it is far superior to a common compilation—if otherwise, it is much more dangerous. Dr. Gregory appears to have hazarded this difficult experiment—and on this account we shall deviate, on the present occasion, from our usual analytical course, to pursue a critical one. Dr. Gregory need not imagine, however, that we seat ourselves in the chair of Aristarchus in order to play the critic. If we notice only what we consider imperfections in his work, it is because our whole journal would not contain what we consider to be sound pathology and judicious practice. And truly, if the frame of Dr. Gregory's mind be constituted as it ought to be, and as we believe it is, he will receive our comments in the spirit of that true philosophy which disdains not to listen to the opinions of others, or accept information from whatever quarter it may be offered. We trust that the tone of this critique will evince the object of it—that of promoting public good, without hurting private feeling.

Dr. Gregory modestly informs the reader that the present volume "has no pretensions to the title of a system of physic, inasmuch as the author neither undertakes to digest all that has hitherto been written about diseases, nor to explain their varied phenomena on any one hypothesis."

> "The object of the author in this work, is to lay before the student of medicine an elementary view of the present state of the theory and practice of medicine, unbiassed by system; and more particularly to delineate those views of pathology which appear to direct the reasonings, and to give a tone to the language of medical writers at the present period." *Preface.*

We much doubt the expression—"unbiassed by system," especially when our author, in the next page, informs us that —"the general design of the volume coincides very nearly with that of Dr. Cullen's First Lines," which is abundantly systematic. The truth we believe is, that Dr. Gregory has somewhat confounded "system" with "theory," though the former is connected principally with arrangement or classification, and does not necessarily mix up any hypothesis with the matter. But notwithstanding our author's professed rejection of hypothesis in the first page of his Preface, we have found him indulging slyly in various dark corners as we proceeded, under the mask of "speculations."

On Dr. Gregory's first class of diseases—fevers, we have few observations to make. Our sentiments respecting fever are now pretty well known, and Dr. G's do not differ materially from those which have been maintained in this Journal for many years past. On some points of doctrine and practice, however, we conceive our author to have been led into inconsistency, if not into error. Thus speaking of the treatment of fever generally, our author remarks that—

"There cannot exist a doubt as to the necessity of blood-letting in the genuine inflammatory fever, the *endemic* of hot climates. The violence of that disease, the rapidity of its progress, and the high degree of arterial excitement which characterize it, call for the adoption of a system of measures, at once powerful and immediate in their effects. On the first attack, therefore, blood is to be taken from the arm to the extent of twenty or thirty ounces, and in a full stream. This it is frequently necessary to repeat in the course of a few hours, the extent of the evacuation being always regulated by the violence of the symptoms, particularly by the degree of head-ache, and the fulness of the pulse. These must be diminished without delay, and, though other means are not to be neglected, it is upon venesection that our chief reliance is to be placed." 60.

Yet a little further on, when speaking of the treatment of yellow fever, such as it has appeared epidemically in the West India Islands, in America, Gibraltar, Cadiz, Malaga, &c. our author hazards the following strange and dangerous assertion.

"The severe head-ache which characterizes the early stages of the disease, naturally suggested blood-letting as a probable means of relief, but experience has proved that, though occasionally, it is not generally beneficial." 103.

How are we to reconcile this contradiction? It is sufficient to say, that Dr. Gregory is a convert to the doctrine of a *contagious and imported* epidemic yellow fever.

In the class of eruptive fevers we observe the following passage:

"On dissection of those who die of small-pox during the first or eruptive stage, the mucous membrane of the bronchia, œsophagus, stomach, and intestines, is found in a state of high inflammation. In those who have died under a load of pustules, the same appearances are sometimes traced, but *pustules* are never met with in the cavity of the abdomen. It appears indeed that the mucous coat of the intestinal tract is unsusceptible of them. Variolous pustules however may be observed in great abundance in the mouth, and along the mucous membrane of the *bronchia*, even as low as their bifurcation. This curious distinction between the two great mucous membranes of the body is probably referable to some difference in their anatomical structure." 127.

We know not how Dr. Gregory came to conclude that the mucous membrane of the intestinal tract was insusceptible of assuming this pustular appearance in small pox; but we can point out to him several sources of information on this head, which, if attended to, would have prevented this position being so strongly laid down. Dr. Clark states that at the *Maison des Enfans* in Paris. The dissections of variolous patients shewed—"inflammation and ulceration of the internal coat of the intestines, *pustular eruptions there*, or on the surface of the peritoneum, &c." *p.* 150. And Mr. Cross and Mr. Hull, in this country, have made the same observation, as may be seen at page 305 of this volume.

In chap. vii. "on laryngeal inflammation," some things have attracted our attention. Dr. Gregory very properly states that "large bleedings are required, and at the onset, they should be pushed so as to produce fainting." But to the precept, that "leeches may be applied to the throat *when the violence of the symptoms has been subdued*," we cannot give our approbation, since we are well convinced that *local* detractions of blood cannot be too soon employed in this formidable disease, where the simultaneous co-operation of remedies should always be preferred to a lingering succession of them. A little farther on Dr. Gregory observes—"as a last resource, some have recommended tracheotomy; but upon the whole, considering the disadvantageous circumstances under which the operation must be performed, it can scarcely be thought adviseable." 234. As a comment on this passage, we shall here introduce a case to which we were an eye-witness, and which for intensity of interest, has no parallel in the annals of surgery. It was originally published in one of the volumes of the *monthly series* of this Journal, which the public most undeservedly suffered to pine in obscurity; consequently its contents are almost entirely unknown to the profession. The following valuable fragment we shall here rescue from oblivion.

"*Case of Cynanche Laryngea, requiring Tracheotomy, and the continued Use of a Canula ever since the Operation.*

"Mr. Price, jeweller, residing in King's Terrace, Southsea, near Portsmouth, about 30 years of age, and previously unaffected with any complaint of the throat or chest, excepting occasional palpitation of the heart, and sense of uneasiness at the epigastrium, had been for a few days, in the beginning of October 1816, affected with *Cynanche Tonsillaris*, which was removed by the common antiphlogistic measures. But after exposure to the night air, the inflammation suddenly returned, and now fell on the glottis and other points of the larynx, causing a dreadful sense of suffocation. The pulse was, at this time, little altered, excepting from the alarm into which the patient was thrown. On examination, the epiglottis was brought into view, and, with the neighbouring parts, appeared inflamed. Leeches were immediately applied round the throat, and he was bled from the arm, *ad deliquium.* Blisters succeeded the leeches; and the most powerful cathartics were exhibited. The vapour of warm water and vinegar was inhaled; and the patient by these means was soon relieved. In three days a copious discharge of *apparently purulent matter* took place from the throat, brought off by hawking and coughing, and giving rise to apprehensions that the previous inflammation had terminated in suppuration or ulceration. This discharge amounted, at one time, to a tea-cup full in twenty-four hours. His breathing was pretty free; but still he had an unpleasant *vox rauca:* he looked ill, and had some pyrexia. The discharge, however, gradually lessened; his appetite returned; and it became evident, that what appeared to be purulent matter, was only a secretion from the inflamed parts.

"When things were in this favourable train, an accidental exposure to a stream of cold air, was almost immediately succeeded by such a difficulty of respiration, as baffled every energetic measure that could be devised for its removal.

"*November 5th,* 1816. In consultation, Dr. Lara, Dr. Denmark, and Dr. Johnson. The respiration is most difficult; eyes prominent; cheeks flushed; lips livid; forehead and breast covered with a cold, clammy perspiration; countenance ghastly, and expressive of unutterable and indiscribable sufferings; both sides of the larynx, to below the cricoid cartilage, tender and swelled; the whole neck tumefied, and red from the sinapisms that had been applied. Death was now rapidly approaching; and it was very doubtful whether effusion in the lungs had not already taken place. It was unanimously agreed that nothing but tracheotomy could save, or, even for a short time, prolong life. It was also feared by two of the three medical attendants, that such organic derangement had already supervened in the larynx, as would render the operation unsuccessful. But this was no solid reason for abstaining from the *anceps remedium.*

"*Operation,* 5, P. M. Dr. Denmark took the knife. An incision through the integuments from the lower edge of the thyroid gland, to within half an inch of the sternum, was made, as nearly as could be guessed, on the line that divides the sterno-hyoid and thyroid muscles of the opposite sides. When these muscles were exposed, however, no such line of division could be perceived, owing, no doubt, to the tumefaction and inflammation of the parts. The incision was therefore carried cautiously through these muscles, in a direction for the trachea. The motion of these muscles, and of those of the neck in general, but of deglutition in particular, was violent, and extremely embarrassing. Several venous and arterial branches were cut, and bled freely, but ceased to bleed without ligature. Anxious to come at the trachea as near the thyroid gland as possible, the right lobe of that body was accidently entered, in consequence of the convulsive action of the muscles. A principal branch of the inferior thyroid artery of that side sprang, and bled profusely. Dr. Denmark plunged a tenaculum as nearly as possible on it, and Dr. Johnson affixed a ligature. The hæmorrhage continued; the ligature was instantly cut away; another plunge was made with the tenaculum, and now the ligature proved successful. It was not thought adviseable to advance farther in that direction, and the enlargement of the wound downwards towards the sternum required caution, lest, in the struggles for breath, some large vessel in that vascular neighbourhood should be divided. At length, after a somewhat tedious dissection, the difficulty of which can only be appreciated by those who see it performed on a living subject gasping and struggling for breath, the trachea was laid bare, at full an inch from the surface. Those who talk about cutting out a piece of a tracheal ring at the bottom of such a cavity as this, while the trachea itself is in constant motion, and the surrounding muscles ever altering their relative positions in the efforts at respiration, have probably never practised it. The tube was slit open, with the point of a double edged scalpel, about a third of an inch, when the hissing of the air, backwards and forwards, became very loud. With some difficulty a flattened silver canula was introduced, when the relief, instantaneously experienced by the unhappy sufferer, may be conceived, but cannot be described. He could hardly be freed from his bloody garments, and propped up in bed, when he fell into a profound sleep! The canula was secured by means of tapes, adhesive straps, &c. and he slept and breathed tranquilly, till nine o'clock at night, when a fit of coughing displaced the tube, and threw him into great distress as well as danger. It was found that the longitudinal opening in the trachea admitted of but a very inadequate ingress and egress of air, when the tube was out; and the introduction of the latter at the bottom of a deep sinus, as the wound was, presented some difficulty. This difficulty was a good deal overcome by the suggestion of Dr. Johnson, viz. that of pushing the end of an elastic gum catheter a little way through the silver canula, so as to make a *blind end*, which readily went into the opening of the trachea, when the gum catheter was immediately withdrawn, and the canula left in. A sound and refreshing night's sleep succeeded this long period of restlessness and misery.

" *November 6th.* This morning the irritation of a foreign body had caused a great secretion of phlegm and mucus throughout the larynx and trachea, and the efforts to cough it up by the mouth and through the canula were very distressing, and required constant attention. The canula was repeatedly displaced. Voice and speech were entirely gone of course; but deglutition was easy, and he this day took some brisk purgative medicine. The countenance had regained much of its pristine serenity ever since the operation; the pulse was regular, but quick; and the symptomatic fever was not considerable. His spirits are now good, and he is constantly writing down and answering questions. By the evening of this day, considerable tumefaction had taken place round the wound; the cavity was consequently rendered deeper; the canula, become too short, was every minute displaced, and would no longer answer. It was always necessary when the tube was removed, to draw one edge of the slit in the trachea apart from the other, by means of a crooked probe or hook, when the patient could breathe with tolerable ease. But the mucous secretion was most tiresome and embarrassing; so much so, that frequently the convulsive struggles to force it up appeared almost decisive of his fate! During the night, a new tube, adapted to the depth of the wound, was many times thrown out, and a surgeon was obliged to be in constant attendance. A strong mercurial fœtor this day was discovered in the breath, from some calomel which he had taken with purgative medicine, during the previous inflammation.

"*7th.* This day a very smart ptyalism developed itself, and the increased flow of saliva from the mouth and fauces added greatly to the patient's other sufferings. The inflammation, however, and tumefaction around the wound were beginning to subside; and, for the first time since the operation, he could articulate a little. When the tube was now displaced by accident or design, the dyspnœa was not quite so distressing as at first; and upon the whole, a gleam of hope broke through the clouds, and gave anticipation of final success. The bowels were kept very free to day, in hopes of checking the ptyalism, which was still profuse; but the countenance was cheerful and serene, excepting during a paroxysm of coughing.

"*8th.* Has passed a good night; the tube was twice displaced by coughing, but easily reinstated. This morning, the artificial passage was closed with the finger, and he breathed by the mouth; but the difficulty was as great as previous to the operation; so that nothing but time has yet been gained. The canula of a common trochar introduced on a bougie that just fills its calibre, answers best now.

"*9th.* Has had a good deal of fever last night; and complained of pain and coldness of the lower extremities. Expectorates much viscid phlegm and mucus, which have a very disagreeable fœtor.

"*10th.* A feverish and restless night; was sometimes delirious; but on taking some active purgatives, he has had four copious stools, and the febrile symptoms have subsided.

"*11th.* Passed a good night; has no fever; breathes comfortably through the tube and mouth together; and could blow out a candle by the air from his mouth alone, of which he was highly proud. Can speak pretty plain. Inhaled warm aqueous vapour through the tube, which he found very pleasant.

"*12th*, *13th*, *14th*. Little or no alteration.

"*15th.* Took out the tube to-day, and closed the wound completely with the finger. The difficulty of breathing was, at first, great; but in a little time it became somewhat easier; and he breathed nearly ten minutes through the natural passage. He is becoming very hoarse, but he sleeps quietly the whole night, and only changes the tube twice in twenty-four hours.

"*20th.* He now eats, drinks, and sleeps well. The tube is every day taken out, and he is made to breathe through the natural passage, as long as he conveniently can; but it is evident that the original obstruction remains. He expectorates some semi-purulent looking mucus, which smells very badly.

"*29th.* The tube has only been changed every second day since last Report. On withdrawing it to-day, he breathed with great difficulty, and we were forced to introduce it with considerable dispatch, as he seemed to be suffocating. There is now every appearance of a tedious or doubtful issue, after all our exertions!

"*December 16th.* Six weeks have now elapsed since the operation. His voice is scarcely audible. He cannot breathe at all, but through the tube. We tried to pass down a curved elastic tube through the rima glottidis, but without success. A finger was passed behind the epiglottis, but the opening into the larynx could not be felt; neither could any morbid structure or ulceration be discovered. Linimentum hydrargyri was directed to be rubbed over the region of the larynx, which evidently brought on a slight ptyalism. An occasional intermission of the pulse is now observable, and he has palpitation of the heart.

"*30th.* Or fifty-six days after the operation, he coughed up through the tube a piece of apparently carious bone of a cancelated structure, and supposed to be a piece of what is termed ossified thyroid or cricoid cartilage. In other respects he is much the same as before.

"*January 1st*, 1817. Last night he felt a piece of bone fall down into the lungs, and has ever since been in a dreadful state of coughing. He feels the piece come up to the tube, but as it cannot get out, it falls back, and keeps him in constant agitation. Something decisive must now be done. The Canula A was therefore quickly constructed, and being much larger in the bore than the one previously in use, besides having its inner orifice turned downwards to facilitate the escape of any solid body, it was forced into the wound with some difficulty. The good effects were soon felt; for shortly afterwards, in a violent convulsive cough, he threw out the piece of bone [or rather calcareous deposition] No. 1, quite across the room where he was sitting! He was immediately relieved. What was very curious, the piece of ossificatfon was larger than the calibre of the tube, and could not be made to enter it afterwards, in any position. After this he threw out several pieces of the same substance: some of which are represented in the plan: but the tube A gave him so much ease, and answered so well, that he wore no other kind for many months afterwards.

"*March 18th*, 1817. Has continued in good health since last Report, and can breathe a few minutes with the tube corked, which is the usual way of exercising the natural passage. He went to London this day, and consulted Mr. Astley Cooper and others, who recommended patience, fumigations, &c. but did not think that any thing in the operation way could be done.

"He remained a month or six weeks in London; and then returned to this place. By corking the tube several times a-day, he at last acquired the power of keeping it so, and of breathing through the mouth for several hours at a time; but he thought it hurt his health; and, latterly, he seldom stops the tube, excepting when he wishes to speak, which he can do plainly enough by placing his finger on the tube. The canula B. is that which he has worn for the last two or three months, and it gives little or no uneasiness.

"*December 5th.* 1817. One year and one month have now elapsed since the operation was performed; and the tube is worn without inconvenience, being merely taken out twice a week to clean, and immediately replaced. His health is as good as at almost any period of his life. There is a slight tumefaction, and also a considerable induration around the wound, from which a discharge proceeds as if from an issue. The actual site or nature of the obstruction in the larynx cannot be ascertained; and nothing but time can determine the result of this case, which stands unique in the annals of surgery."

Three years have passed away since the above case was published, and the patient still lives and breathes through the tube. He is otherwise in good health. We believe that we need not comment farther on Dr. Gregory's condemnation of tracheotomy, especially as it is well known to the profession, that several other cases of bronchotomy have within these few years, terminated successfully, and saved the lives of the patients.

Upon diseases of the heart and of the liver, Dr. Gregory is less copious than we could have wished. In the section on dysentery we have observed a good deal that we cannot entirely approve. Dr. Gregory allows that, in all severe cases of this disease, there is inflammation and much morbid irritability of the mucous membrane of the intestines. Yet strange enough, he says that—"the employment of *purgatives* constitutes the most important part of the treatment." We would be glad to know on what principle the irritation of purgatives is superadded to the irritation already existing in the mucous membrane?—But leaving theory aside, it is well known to all those who have treated dysentery, at the bedside, that the practice of purgation is exceedingly distressing in severe cases—while, in mild ones it keeps up the disease much longer than is generally necessary for a cure, besides adding greatly to the sufferings of the patient.

"In hot climates, the exhibition of mercury, pushed so as to produce salivation, has been supposed by some, to be an effectual method of putting a check to the advances of the disease. The testimonies in favour of this practice are certainly very strong, but at the same time it is to be observed, that we have no reason to believe that a vigorous and well regulated employment of the means already recommended, is less efficacious in hot climates than we find it in our own." 313.

Now, from some little experience, we are enabled to assure Dr. Gregory, that instead of there being *no reason* to believe that those means which he enumerates would be less efficacious in tropical climates than the mercurial treatment, there is *direct proof* of their inefficacy.

The foregoing are the principal objections which we have to urge against the work before us; and truly they are very few. For the rest, it appears to us, in most places, to be very neatly compiled or composed; and, upon the whole, is very creditable to the good sense and judgment of the author.

